# The crosstalk between autophagy and apoptosis was mediated by phosphorylation of Bcl-2 and beclin1 in benzene-induced hematotoxicity

**DOI:** 10.1038/s41419-019-2004-4

**Published:** 2019-10-10

**Authors:** Yujiao Chen, Wei Zhang, Xiaoli Guo, Jing Ren, Ai Gao

**Affiliations:** 1 Department of Occupational Health and Environmental Health, School of Public Health, Capital Medical University, Beijing, 100069 China; 2 Beijing Key Laboratory of Environmental Toxicology, Capital Medical University, Beijing, 100069 China

**Keywords:** Apoptosis, Autophagy, Molecular biology

## Abstract

Increasing evidence suggested that benzene exposure resulted in different types of hematological cancer. Both autophagy and apoptosis were reported to play vital roles in benzene toxicity, but the relationship between autophagy and apoptosis remain unclear in benzene-induced hematotoxicity. In this study, the toxic effect of benzene on autophagy and apoptosis in benzene-exposed workers and in vitro were verified. Results showed that benzene metabolite (1, 4-benzoquinone, 1, 4-BQ) dose-dependently induced autophagy and apoptosis via enhancing phosphorylation of Bcl-2 and beclin1. Finally, we also found that the elevated ROS was in line with enhancing the phosphorylation of Bcl-2 and beclin1 which contributed to 1, 4-BQ-induced autophagy and apoptosis. Taken together, this study for the first time found that the effect of 1, 4-BQ on the crosstalk between autophagy and apoptosis were modulated by the ROS generation via enhancing phosphorylation of Bcl-2(Ser70) and phosphorylation of beclin1(Thr119), which offered a novel insight into underlying molecular mechanisms of benzene-induced hematotoxicity, and specifically how the crosstalk between autophagy and apoptosis was involved in benzene toxicity. This work provided novel evidence for the toxic effects and risk assessment of benzene.

## Introduction

Benzene is widely used as an essential industrial material, and is also commonly presented in oil, gasoline vapors, wood-burning and cigarette smoke^1,2^. With the widespread prevalence of benzene, benzene is also known as a common air pollutant in the environment^3^. During industrial production, occupational exposure to benzene occurs in rubber production plants, shoe manufacturing and printing factories mainly via inhalation^4,5^. Exposure to benzene can cause various health hazards, including hematotoxicity^6,7^, aplastic anemia^8^, and human leukemogen^9^. At present, attention on toxic effect of low-dose benzene exposure has been gain. Therefore, it is necessary to explore the effects and mechanisms which evaluates the health effect of benzene exposure.

Various biological processes could induce the benzene toxicity, such as oxidative stress, apoptosis and autophagy. Although recent studies have shown that oxidative stress is widely recognized as a major factor of benzene-induced hematotoxicity, the cause of benzene toxicity has not been fully elucidated^10–12^. The studies have reported benzene activated reactive oxygen species (ROS) in the workers of benzene exposure^11–13^. However, the exact molecular mechanisms underlying the effect of oxidative stress on benzene-induced hematotoxicity are still not clear.

A recent reported revealed that the activation of oxidative stress resulted in apoptosis^14^. Besides, apoptosis was reported to be involved in benzene-induced toxicity^11,12,15^. Several studies revealed that autophagy was induced under benzene exposure and suggested that autophagy played a key role in benzene toxicity^15,16^. Recently, there has been growing interest in the relationship between autophagy and apoptosis. However, it is still not clear whether benzene regulated the crosstalk between autophagy and apoptosis.

Apoptosis and autophagy are different types of cell death, and there are various molecular mechanisms involved in the regulation of the crosstalk between autophagy and apoptosis, such as the expression of beclin1 and Bcl-2, beclin1-Bcl2 complex and the modification of beclin1 and Bcl-2^17,18^. Among them, the interaction and modification of beclin1 and Bcl-2 played a key role in modulating the crosstalk between autophagy and apoptosis. Moreover, previous findings showed that Bcl-2 regulated autophagy via beclin1^19,20^, and Bcl-2 phosphorylated resulted in dissociating from beclin1 and induction of autophagy^21,22^. However, whether the expression, interaction and modification of beclin1 and Bcl-2 are involved in the crosstalk between benzene-induced autophagy and apoptosis remained unclear.

Hence, we proposed that benzene stimulated ROS generation and then induced the oxidative stress that led to the phosphorylation of beclin1 and Bcl-2, which accelerated the activation of autophagy and apoptosis causing benzene-induced hematotoxicity. This study firstly measured the effects of benzene on the crosstalk between autophagy and apoptosis. Then, the relationship between autophagy and apoptosis was detected. Further, the effects of beclin1-Bcl2 complex on benzene-induced autophagy and apoptosis was evaluated. Eventually, the autophagy inhibitor 3-Methyladenine (3-MA) and the apoptotic inhibitor (Z-VAD-FMK) were employed to deeply investigate the molecular mechanisms by which benzene effected the crosstalk between autophagy and apoptosis. This study for the first time revealed that benzene-induced hematotoxicity via enhancing phosphorylation of Bcl-2 and phosphorylation of beclin1, which contributed to the crosstalk between autophagy and apoptosis. This study aimed to explore exposure to benzene could be a potential hazardous effect on hematotoxicity.

## Materials and methods

### Study population

We selected 140 workers randomly, and the benzene concentration of 70 workers was negligible, while 70 workers were known to occupationally exposed to benzene. Participants were required to fill a consent form and answer a questionnaire, including life-style, demographic and occupational information including gender, age, drinking, smoke, medications, and family history of health status. This study was approved by the Committees for Ethical Review of Research involving Human Subjects of Capital Medical University.

### Exposure assessment

Our study monitored individuals’ exposure airborne benzene for 5 h of a working day. Trans, trans-muconic acid (t, t-MA) and S-phenylmercapturic acid (S-PMA) are the urinary metabolites, which were also measured by LCP–MS (Agilent 7700x, USA) in urine samples collected from study participants.

### Routine blood and ALT

Hospital automated blood analyzer (Brand, Germany) was used to measure routine blood, which containing white blood cell (WBC), neutrophil (NEUT), red blood cell (RBC), platelet (PLT), lymphocytes (LYMs) and haemoglobin (HGB) and alanine transaminase (ALT).

### ELISA assays

ELISA assays were performed to determine the level of oxidative, autophagy and apoptosis according to the manufacturer’s instructions. Then culture supernatants and the serum were harvested, centrifuged, and placed. The oxidative stress-related protein (MDA, 8-OHdG, 8-iso-PGF2a, and NQO1) and autophagy-associated and apoptosis-associated protein (Bcl-2, beclin1, p62) were used to determine oxidative, autophagy, and apoptosis present in the culture supernatants and serum. All assays were performed in duplicate and repeated three times.

### The TEM observation

The normal human lymphocyte line (AHH-1) was provided by the National Institute for Radiological Protection, China CDC (Chinese Center for Medical Response to Radiation Emergency). After incubating for 24 h with 20 μM 1,4-BQ, cells were washed 3 times with PBS and harvested by centrifugation for 5 min at 1200 rpm. Then the supernatant was washed 3 times with PB and fixed with 1% citric acid for 4 h. The cell sample was dehydrated in a series of ethanol, and finally embedded in an epoxy resin. Ultrathin sections were obtained using an ultramicrotome and then stained with aqueous uranyl acetate and aqueous lead citrate. After that, cell samples were imaged by a transmission electron microscope (TEM).

### mRFP-GFP-tagged LC3

Cells were transfected with a fluorescent mRFP-GFP-tagged LC3-expressing virus (Genechem, GPL2001A) according to the manufacturer’s instructions. Cells were transfected for 72 h. The cells then were detected after 1, 4-BQ treating for 24 h. GFP and mRFP expression was visualized with a confocal microscope (Leica Microsystems, Germany). Autophagic flux was detected via analyzing the punctate pattern of GFP and mRFP.

### Immunofluorescence and confocal microscopy

Cells were exposed to 1, 4-BQ for 24 h, then fixed with 4% paraformaldehyde for 30 min, washed 3 times with PBS, and permeabilized with 0.5% TritonX-100 in PBS for 10 min at room temperature. Then, the cells were blocked with 10% normal goat serum for 1 h. After that, cells were incubated with rabbit anti-LC3B, or simultaneously incubated rabbit anti-Bcl-2 antibody and mouse anti-beclin1 antibody overnight at 4 °C followed by secondary antibody for 1 h at room temperature. Finally, cells were counterstained with DAPI and imaged with a confocal microscope.

### RNA isolation and quantitative real-time PCR (qRT-PCR)

Total RNA was extracted with Column Blood RNAOUT (TIANDZ, China) according to the manufacturer’s protocol. To determine mRNA levels, RevertAid First strand cDNA (Thermo Fisher Scientific, USA) was synthesized using 1 µg of total RNA in 20 µL reverse transcriptase reaction mixture according to the manufacturer’s protocol. Then quantitative real-time polymerase chain reaction (QRT-PCR) was performed on Bio-Rad (CFX96TM optics Module) using SYBR Green (Thermo Fisher Scientific, USA).

### Co-immunoprecipitation

Cell extract (100 mg) was precleared with Protein-G agarose, then incubated at 4 °C overnight with beclin1 and Bcl-2 antibody with constant rotation. Protein G-sepharose beads were prewashed three times in immunoprecipitation buffer, 0.5% Triton X-100 for 15 min and then incubated at 4 °C for 6 h with the protein/antibody mixture with constant rotation. The precipitant was collected by centrifugation at 10,000 × *g* for 1 min and washed three times with immunoprecipitation buffer to remove nonspecifically bound proteins. The washed beads were suspended in sodium dodecyl sulfate-polyacrylamide gel electrophoresis (SDS–PAGE) loading buffer (30 ml/tube). Beads were removed by centrifugation at 10,000 × *g* for 1 min and the supernatant was analyzed by SDS-PAGE and western blotting.

### Western blotting

Total cellular protein lysates were prepared by lysing cells with a protease inhibitor cocktail and a phosphatase inhibitor cocktail. Equal amounts of total proteins were separated for phos-beclin1(p-Thr119), phos-Bcl2(p-Ser70), SQSTM1, beclin1, Bcl-2, LC3B, and Caspase-3 detection. Actin was used as the protein loading control. Experiments were performed for at least third times and a representative experimental result was shown. Grayscale analysis of proteins was quantified with Imaging J.

### Statistical analysis

Statistical analysis was performed by the Statistical Package for the Social Sciences (SPSS) software version 17.0. The Kolmogorov-Smirnov tests were used to check Normality Distributions of all variables. The differences between the two groups were analyzed by independent-sample t tests. And the data were presented by mean ± SD values. The result was considered to be statistically significant when *p*-values in 2 sides < 0.05.

## Results

### The concentration of airborne benzene and urinary benzene metabolites levels

In this population-based study, 140 workers were recruited, including 70 non-known benzene workers and 70 benzene-exposed workers. Additionally, smoking, life style and alcohol consumption between the two groups were matched.

The median of air benzene concentrations was 0.050 mg/m^3^ and 2.639 mg/m^3^ in control and benzene exposure group, respectively. As shown in Supplemental material, Supporting Table 1s, there was a statistically significant difference of airborne benzene concentration in control and benzene exposure group.

Supplemental material, Supporting Table 2s showed that the levels of urinary metabolites (S-PMA and t, t-MA) in benzene exposure group were higher than that seen in controls. However, no significant difference between control and benzene exposure group in terms of urinary metabolites (S-PMA and t, t-MA) was found in all subjects.

### Oxidative stress injury, autophagy, and apoptosis were correlated with benzene exposure

**Fig. 1:**
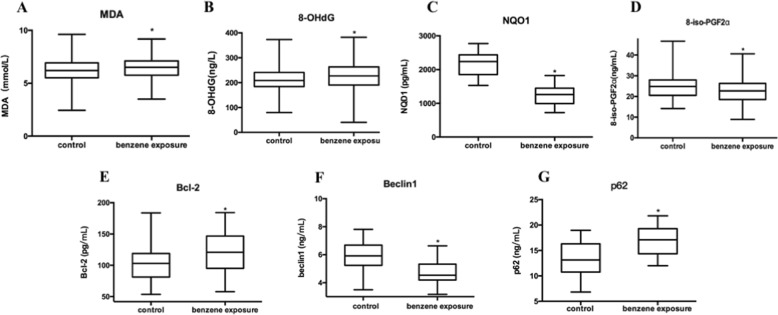
Oxidative stress, autophagy and apoptosis were correlated with benzene exposure. **a**–**d** The level of oxidative stress was measured using ELISA assay. The indicators of oxidative stress, including MDA, 8-OHdG, NQO1 and 8-iso-PGF2a, were measured in control (*n* = 70) and benzene exposure group (*n* = 70). *p < 0.05, compared to controls. **e**–**g** The expression of Bcl-2, beclin1 and p62 was measured by ELISA assay. Data are represented in the form of mean ± SD. *p < 0.05, compared to control group

It was reported that oxidative stress is a main mechanism of benzene-induced toxicity^7,10,23^. MDA, 8-OHdG, NQO1 and 8-iso-PGF2a, reflecting the level of oxidative stress which triggered by low-dose benzene exposure, were detected using ELISA. Figure 1a, b showed that MDA and 8-OHdG had a rise trend, and there was statistically difference between two groups. 8-iso-PGF2a and NQO1 in benzene exposure group was higher than in control group (Fig. 1c, d). These results indicated that benzene exposure led to oxidative stress injury.

**Fig. 2:**
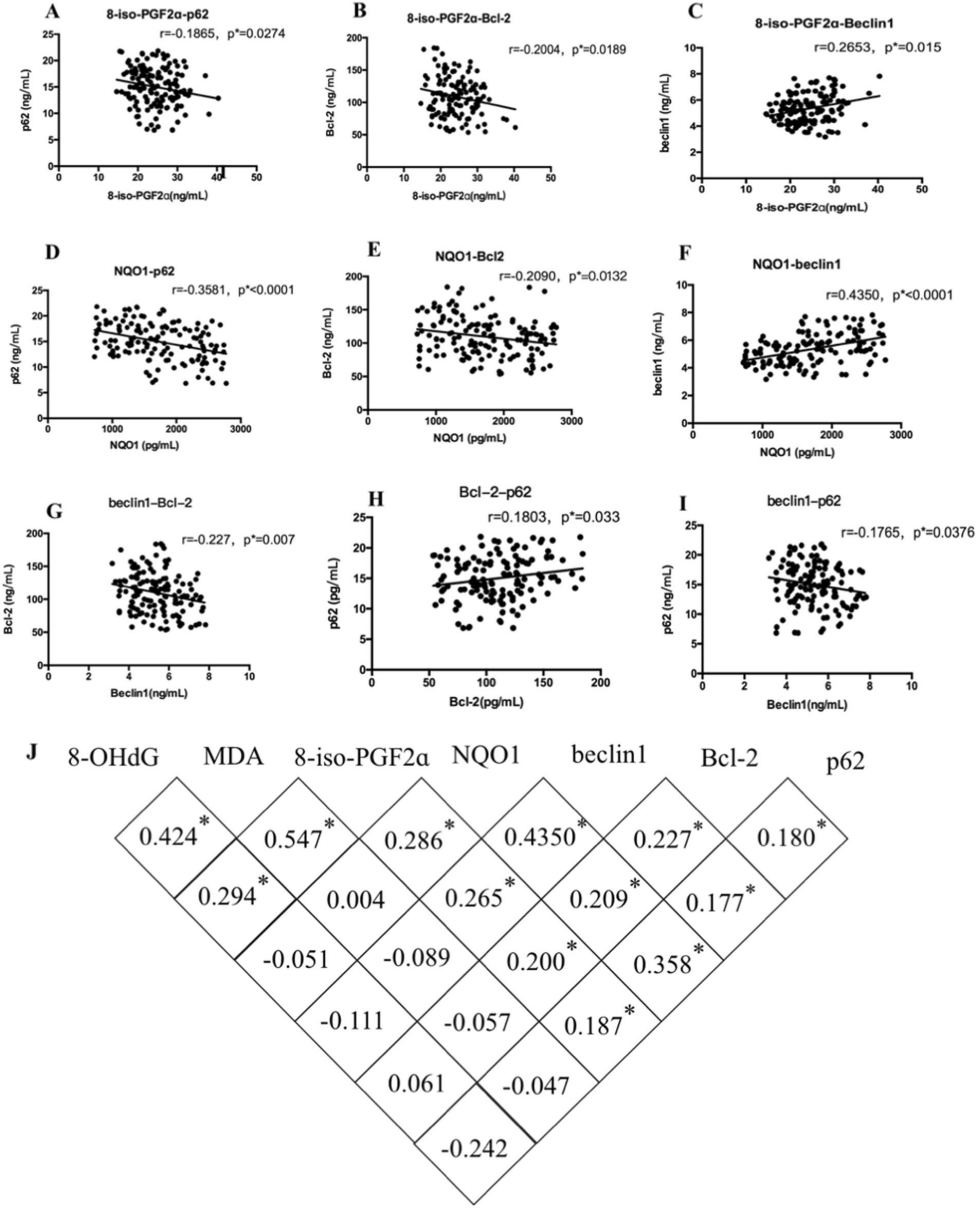
Oxidative stress was significantly correlated to benzene-induced autophagy and apoptosis. **a**–**j** The correlations between oxidative stress, autophagy and apoptosis were analyzed using correlation analysis. Pearson’s correlation among oxidative stress, autophagy-associated and apoptosis-associated proteins was respectively calculated. Data are represented in the form of mean ± SD. *p < 0.05 compared to control group

In order to investigate the effect of autophagy and apoptosis on benzene-induced hematotoxicity, Bcl-2, beclin1 and p62 was measured by ELISA assay. Bcl-2 and p62 in benzene exposure group was higher than in control group, while beclin1 in benzene exposure group was lower than in control group (Fig. 1e–g). To establish the relationship among oxidative stress injury, autophagy and apoptosis triggered by benzene exposure, the correlated analysis was performed. As shown in Fig. 2a–f, j, NQO1 and 8-iso-PGF2a were highly correlated with the autophagy-associated protein (beclin1 and p62) and apoptosis-associated protein (Bcl-2). The results of Supplemental material, Supporting Fig. 1s showed MDA and 8-OHdG were not associated with autophagy and apoptosis, suggesting that the decrease of NQO1 triggered by oxidative stress was closely correlated with benzene-induced autophagy and apoptosis. In addition, Fig. 2g–j demonstrated that Bcl-2 expression was closely related to beclin1 and p62 expression. The results illustrated that benzene-induced abnormal autophagy and apoptosis were closely related to the activation of oxidative stress.

### The induction of autophagy and apoptosis in benzene-exposed group was correlated with benzene-induced hematotoxicity

**Fig. 3:**
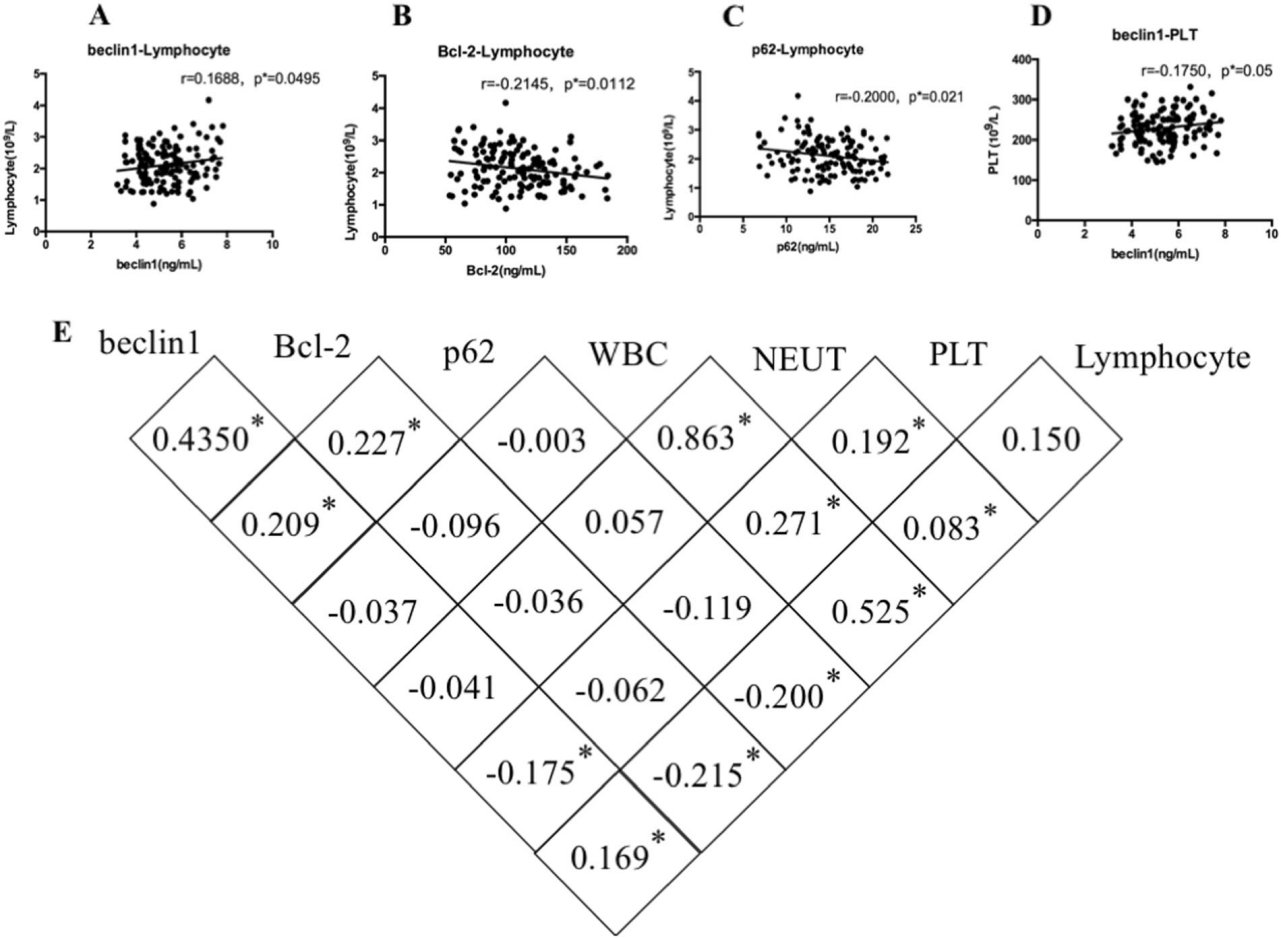
The induction of autophagy and apoptosis in benzene-exposed group was correlated with benzene-induced hematotoxicity. **a**–**e** The correlations between autophagy-associated and apoptosis-associated proteins and blood clinical parameters which reflecting benzene-induced hematotoxicity

Blood clinical parameters reflects hematotoxicity, PLT, and LYM manifested a significant reduction in benzene exposure group compared with control group (Supplemental material, Supporting Fig. 1s). To further investigate the exact mechanism of benzene-induced hematotoxicity, the relationship between cell death-associated (autophagy and apoptosis) protein and blood clinical parameters was analyzed by correlation analysis in the population-based study (Fig. 3e). Interestingly, we found that the protein of beclin1, Bcl-2, and p62 were both closely related to the mount of LYMs. Population-based results were consistent with results in vitro, which used LYMs to explore mechanisms in vitro(Fig. 3a–c). The correlation analysis also showed that beclin1 was closely related to PLT (Fig. 3d), but no other correlations were found in this study (Fig. 2sa–h). Therefore, we concluded that benzene might induce hematotoxicity via modulating the crosstalk between autophagy and apoptosis.

### 1, 4-BQ induced autophagy and apoptosis via the activation of oxidative stress

**Fig. 4:**
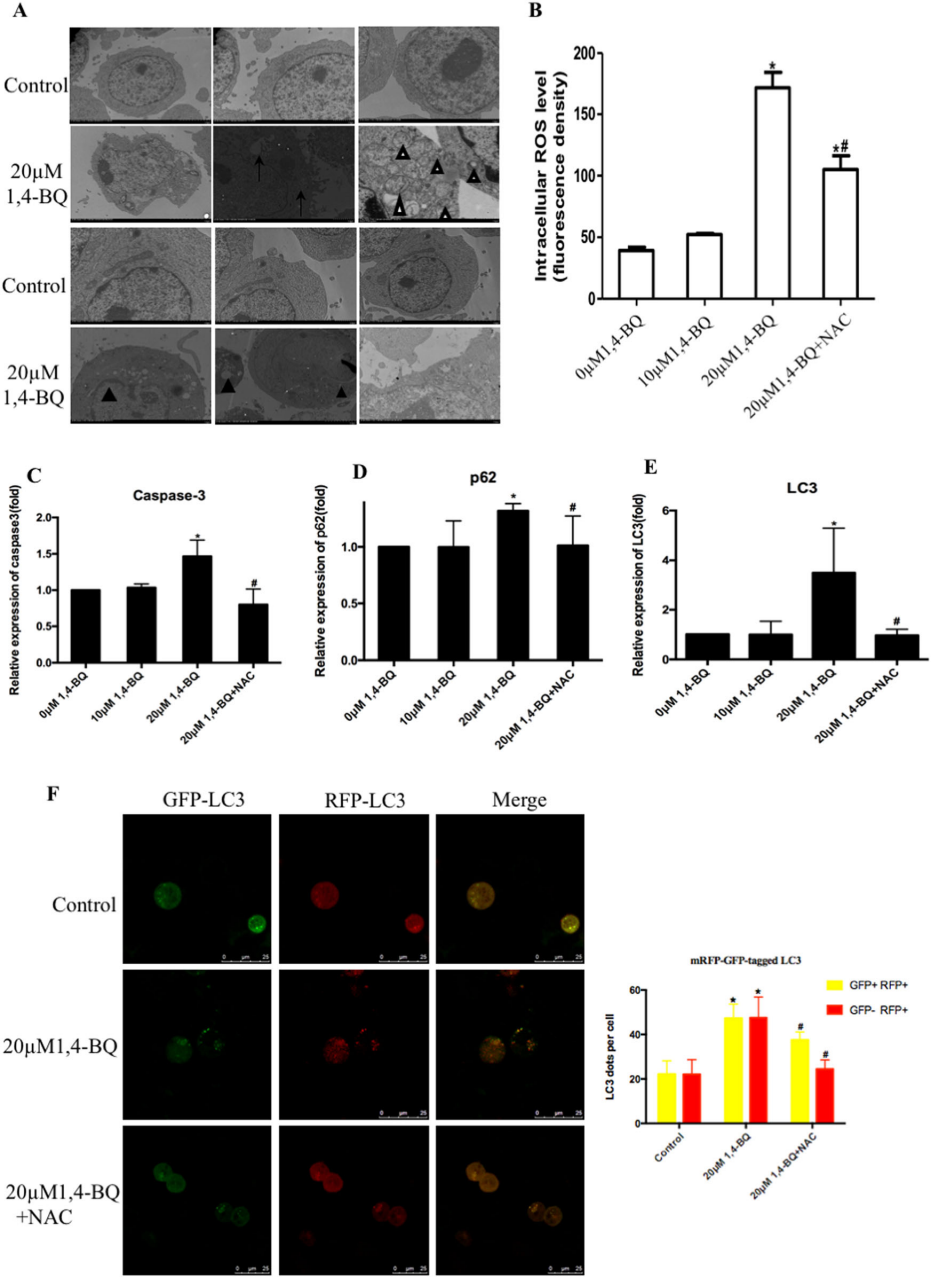
1, 4-BQ induced autophagy and apoptosis via activating oxidative stress. **a** The images of TEM showed that 1, 4-BQ induced autophagy and autophagosome accumulation. White arrow, double-membrane autophagosome (White triangle) and single-membrane autolysosome (Arrows). TEM ultrastructural analysis showed that 1, 4-BQ activated apoptosis and the formation of apoptotic body. The chromatin margination, nuclear fragmentation and apoptotic body were observed (Black triangle). **b** The level of oxidative stress was measured by fluorescent microplate reader. **c**–**e** The toxic effect of 1, 4-BQ on the expression of autophagy- and apoptosis-associated genes was investigated by qRT-PCR. **f** After treating 1, 4-BQ and oxidative stress inhibitor (NAC), autophagy evaluated by mRFP-GFP-LC3 virus

In order to confirm the activation of autophagy and apoptosis, TEM, Western bolt and ELISA assays were also performed to confirm the effect of 1, 4-BQ on autophagy and apoptosis in the AHH-1 cells. TEM assay was a common method for detecting the activation of autophagy and apoptosis. The untreated cells were morphologically normal, while there were many autophagic vacuoles and double-membrane autophagosomes in the 1, 4-BQ-treated cells (Fig. 4a). We also found that cells showed apoptotic-like ultrastructural changes such as chromatin margination, cytoplasmic vacuolization, nuclear fragmentation and apoptotic body formation after treating with 1, 4-BQ (Fig. 4a). These representative images of TEM proved the significant evidence that 1, 4-BQ activated autophagy and apoptosis.

**Fig. 5:**
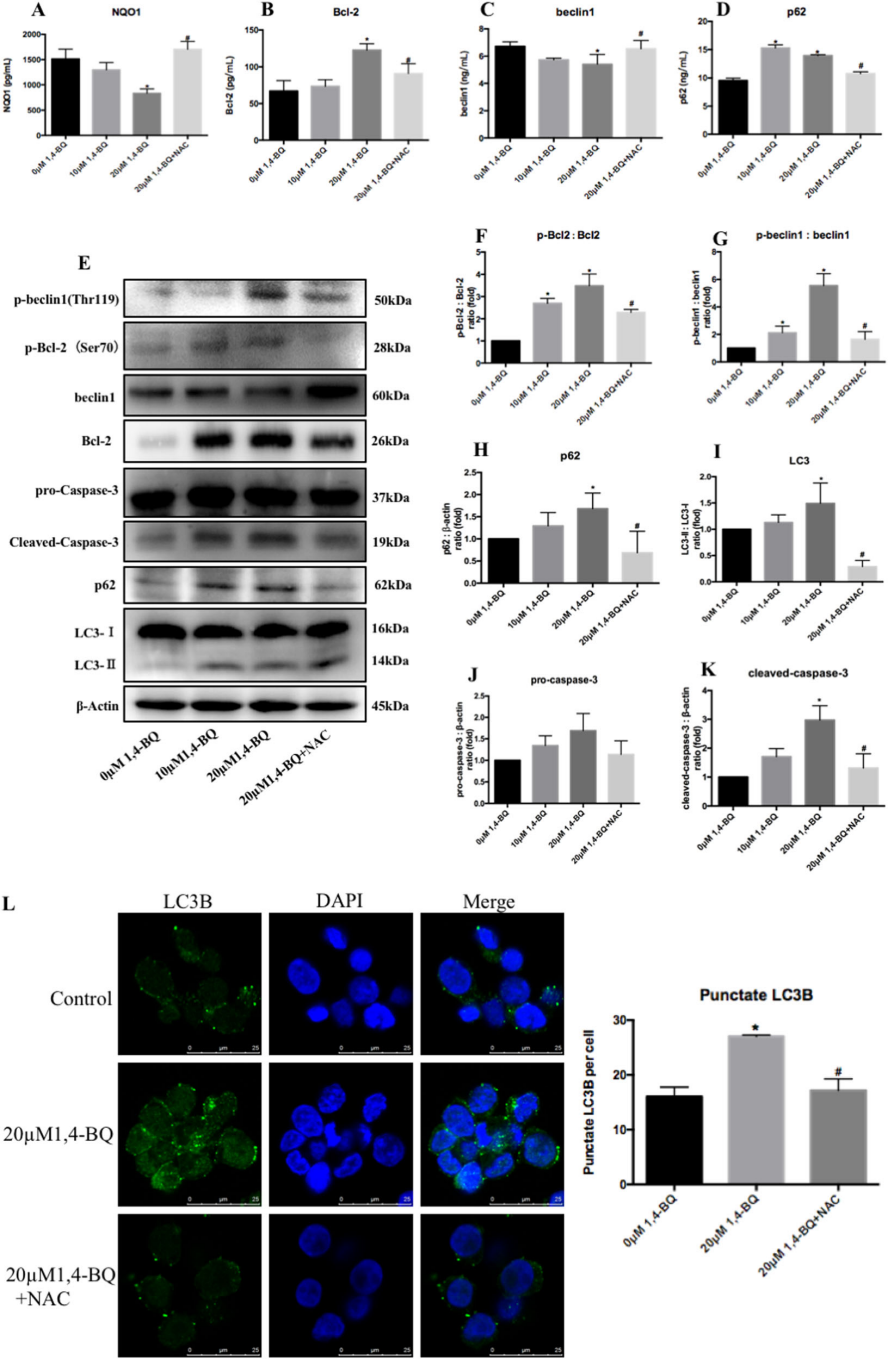
1, 4-BQ induced autophagy and apoptosis by enhancing phosphorylation of Bcl-2 at Ser70 site and phosphorylation of beclin1 at Thr119 site. **a**–**d** The NQO1, Bcl-2, beclin1, and p62 were measured using ELISA assay after oxidative stress inhibitor(NAC) treatment. **e**–**k** Western blotting was used to analyze the level of phosphorylation of Bcl-2 at Ser70 site, phosphorylation of beclin1 at Thr119 site and autophagy- and apoptosis-associated proteins. Data are represented in the form of mean ± SD. *p < 0.05 compared to control group. ^#^*p* < 0.05 compared to 20 μM 1,4-BQ-treated group. **l** The image of immunofluorescence was analyzed by Image J. Data are represented in the form of mean ± SD. *p < 0.05 compared to control group. ^#^*p* < 0.05 compared to 20 μM 1,4-BQ-treated group

To get a deeper insight into the underlying mechanism of autophagy and apoptosis which were triggered by benzene metabolite 1, 4-BQ, the level of intracellular oxidative stress was measured using the DCFH-DA probe. As shown in Fig. 4b, the level of oxidative stress gradually elevated in AHH-1 cells with increasing concentrations of 1, 4-BQ. Thus, the oxidative stress inhibitor NAC reversed 1, 4-BQ-activated oxidative stress (Fig. 4b). To investigate the effect of ROS on 1, 4-BQ-induced autophagy and apoptosis, the change of 1, 4-BQ-induced autophagy and apoptosis was detected using qRT-PCR, Western blotting and ELISA assay after treating with oxidative stress inhibitor NAC. Administration of NAC along with 1, 4-BQ reversed the increase of autophagy-associated and apoptosis-associated genes (p62, LC3 and caspase-3) (Fig. 4c–e). Thus, the effect of NAC on autophagy were measured using mRFP-GFP-tagged LC3 which monitored autophagosomes and autolysosomes to further examine the change of autophagic flux (Fig. 4f). We found that the numbers of puncta corresponding to autophagosomes (GFP+ RFP+) and autolysosomes (GFP− RFP+) increased after 1, 4-BQ-teated cells. NAC treatment reversed the increase of autophagosomes (GFP+ RFP+) and autolysosomes (GFP- RFP+) induced by 1, 4-BQ. As shown in Fig. 5a, the expression of NQO1 was measured by ELISA assay. The NQO1 dose-dependently decreased, and NAC treatment effectively reversed the reduction of NQO1 which induced by 1, 4-BQ. Figure 5b–d showed that the protein expression of Bcl-2, p62 and beclin1 reversed after NAC treatment which revealed that NAC reversed 1, 4-BQ-induced autophagy and apoptosis. Moreover, in the NAC + 20 μM 1, 4-BQ group, NAC treatment further inhibited the p62 and LC3I/II conversion which were markers of autophagy activation (Fig. 5e, h–j). Thus, the effect of NAC on apoptosis were measured using the level of cleaved-caspase-3 (Fig. 5k). Besides, the immunofluorescence analysis further revealed that LC3B puncta increased in 1, 4-BQ-treated cells. And the increase of punctate LC3B induced by 1, 4-BQ was reversed after NAC treatment (Fig. 5l). Therefore, we concluded that 1, 4-BQ activated autophagy and induced apoptosis by activating oxidative stress.

### 1, 4-BQ activated autophagy and induced abnormal apoptosis via enhancing Bcl-2 Ser70 phosphorylation and beclin1 Thr119 phosphorylation which triggered by ROS

To evaluate the mechanism of oxidative stress on benzene-induced autophagy and apoptosis, we therefore investigated whether phosphorylation of Bcl-2 and phosphorylation of beclin1 caused 1, 4-BQ-induced autophagy and apoptosis. The results showed Bcl-2 Ser70 phosphorylation and beclin1 Thr119 phosphorylation obviously increased after 1, 4-BQ treatment. The results in Fig. 5e–g presented that the level of phosphor-Bcl-2 (Ser70) and phosphor-beclin1 (Thr119) elevated gradually with increasing concentrations of 1, 4-BQ. After treating with NAC, the phosphor-Bcl-2 (Ser70) and phosphor-beclin1 (Thr119) was inhibited. In addition, the immunofluorescence analysis of phosphor-beclin1 (Thr119) also showed that the increase of phosphor-beclin1 (Thr119) induced by 1, 4-BQ was reversed after treating with NAC (Fig. 4s). The results demonstrated that ROS activated autophagy and apoptosis via enhancing phosphorylation of Bcl-2 at Ser70 site and phosphorylation of beclin1 at Thr119 site.

### 1, 4-BQ modulated the crosstalk between autophagy and apoptosis through the dissociation of beclin-Bcl2 complex but not beclin1 expression

**Fig. 6:**
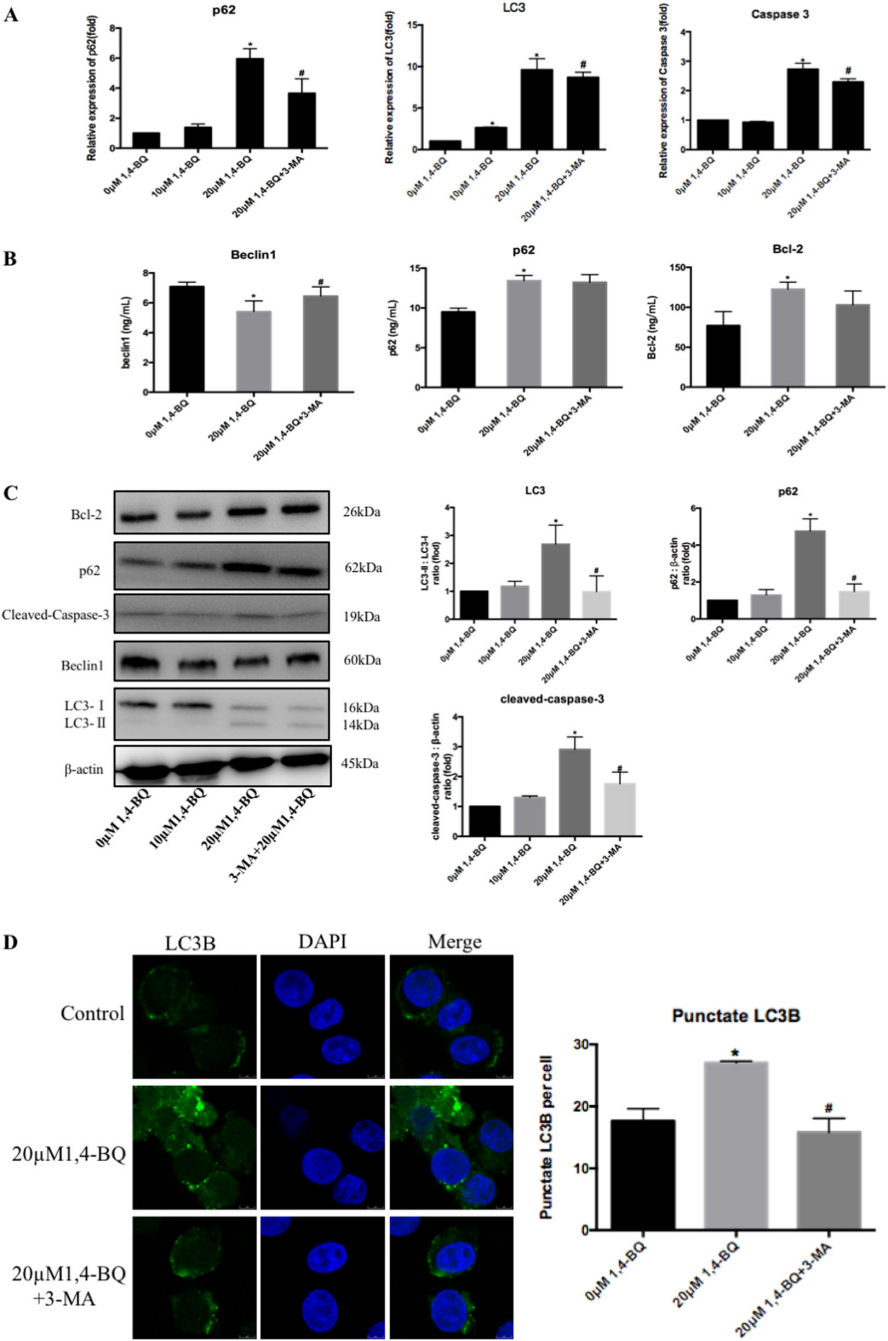
1, 4-BQ-induced autophagy promoted abnormal apoptosis. **a** After treating with autophagy inhibitor (3-MA), the toxic effect of 1, 4-BQ on the expression of autophagy- and apoptosis-associated genes was investigated by qRT-PCR. **b** The Bcl-2, beclin1 and p62 were measured using ELISA assay after autophagy inhibitor (3-MA) treatment. **c** Western blotting was used to analyze the level of proteins which were related to autophagy and apoptosis. **d** After treating with autophagy inhibitor (3-MA), LC3B puncta was analyzed by image J. Data are represented in the form of mean ± SD. *p < 0.05 compared to control group. ^#^*p* < 0.05 compared to 20 μM 1,4-BQ-treated group

**Fig. 7:**
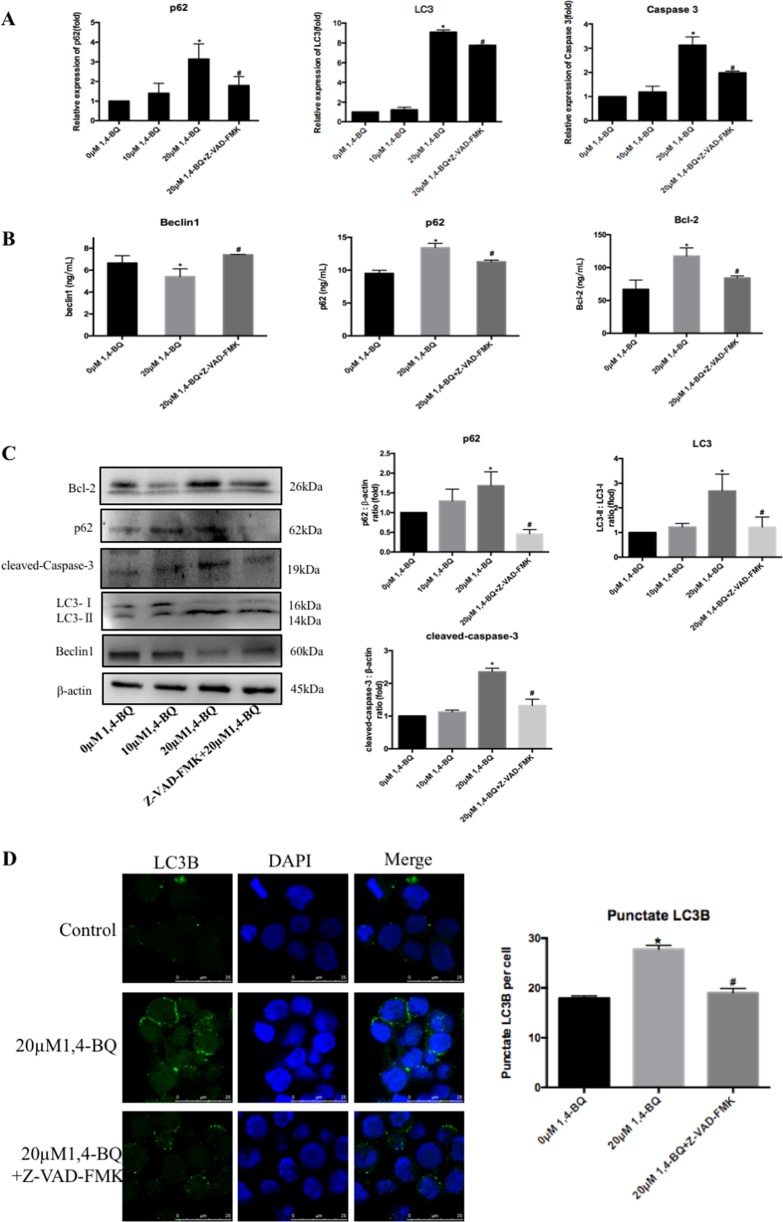
1, 4-BQ-induced apoptosis in turn enhanced autophagy. **a** After treating with apoptosis inhibitor (Z-VAD-FMK), the toxic effect of 1, 4-BQ on the expression of autophagy- and apoptosis-associated genes was investigated by qRT-PCR. **b** The Bcl-2, beclin1, and p62 were measured using ELISA assay after apoptosis inhibitor (Z-VAD-FMK) treatment. **c** Western blotting was used to analyze the level of proteins which were related to autophagy and apoptosis. **d** After treating with apoptosis inhibitor (Z-VAD-FMK), LC3B puncta was analyzed by image J. Data are represented in the form of mean ± SD. *p < 0.05 compared to control group. ^#^*p* < 0.05 compared to 20 μM 1, 4-BQ-treated group

Autophagy and apoptosis are cellular mechanisms for benzene-induced hematotoxicity^16^. To investigate the crosstalk between autophagy and apoptosis on 1, 4-BQ-induced hematotoxicity, the level of autophagy and apoptosis were measured after autophagy inhibitor 3-MA and apoptosis inhibitor Z-VAD-FMK. Administration of 3-MA along with 1, 4-BQ regulated the expression of autophagy-associated and apoptosis-associated genes (p62, LC3 and caspase-3) (Fig. 6a), and inhibited autophagy-associated and apoptosis-associated protein (beclin1, Bcl-2, LC3I/II conversion, p62 and cleaved-caspase-3) (Figs. 6b, c and 5s), indicating that the inhibition of autophagy decreased the level of apoptosis. Thus, apoptosis inhibitor Z-VAD-FMK can reverse the expression of autophagy-associated and apoptosis-associated genes (p62, LC3 and caspase-3) (Fig. 7a) and autophagy-associated and apoptosis-associated protein (beclin1, Bcl-2, LC3I/II conversion, p62 and cleaved-caspase-3) (Figs. 7b, c and 5s), indicating that the inhibition of apoptosis suppressed the level of autophagy. The images of immunofluorescence showed that 3-MA and Z-VAD-FMK decreased the LC3B puncta numbers in the 1, 4-BQ-treated cells (Figs. 6d and 7d). Therefore, we concluded that there was crosstalk between 1, 4-BQ-induced autophagy and apoptosis.

**Fig. 8:**
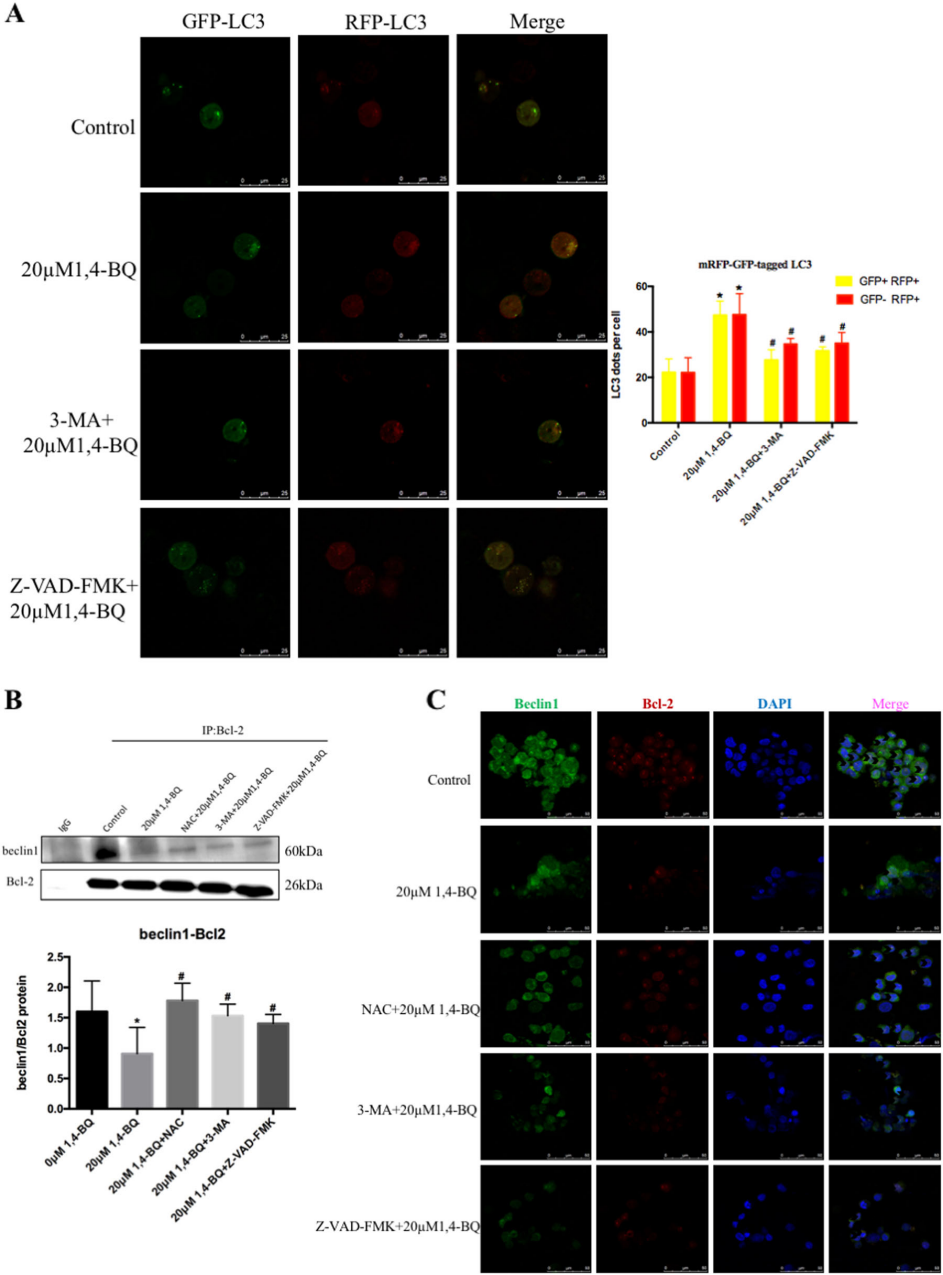
1, 4-BQ mediated the crosstalk between autophagy and apoptosis, and 1, 4-BQ caused dissociation of beclin1-Bcl2 complex. **a** After treating 3-MA and Z-VAD-FMK, 1, 4-BQ-induced autophagy evaluated by mRFP-GFP-LC3 virus. **b** Interaction of beclin1 and Bcl-2 was detected by co-immunoprecipitation. **c** The localization of beclin1 and Bcl-2 in cells is detected by immunofluorescence. Data are represented in the form of mean ± SD. *p < 0.05 compared to control group. ^#^*p* < 0.05 compared to 20 μM 1, 4-BQ-treated group

To investigate whether apoptosis in turn promoted autophagy which activated by 1, 4-BQ, cells were transfected with a tandem monomeric red fluorescent protein (mRFP)-green fluorescent protein (GFP)-LC3 virus to estimate the change of autophagy flux after Z-VAD-FMK and 1, 4-BQ treatment (Fig. 8a). Compared with 1, 4-BQ-treated group, the results of mRFP-GFP-tagged LC3 showed that autophagosomes (GFP+ RFP+) and autolysosomes (GFP− RFP+) in the 1, 4-BQ + 3-MA group obviously decreased (Fig. 8a). Thus, the results of mRFP-GFP-tagged LC3 found that apoptosis inhibitor Z-VAD-FMK obviously reduced 1, 4-BQ-induced autophagosomes (GFP+ RFP+) and autolysosomes (GFP− RFP+) (Fig. 8a). The results demonstrated that 1, 4-BQ upregulated the conversion of LC3-II from LC3-I through upregulation of autophagy rather than blocking autophagosome-lysosome fusion. Therefore, these results revealed that there was crosstalk between autophagy and apoptosis which were activated by 1, 4-BQ.

**Fig. 9:**
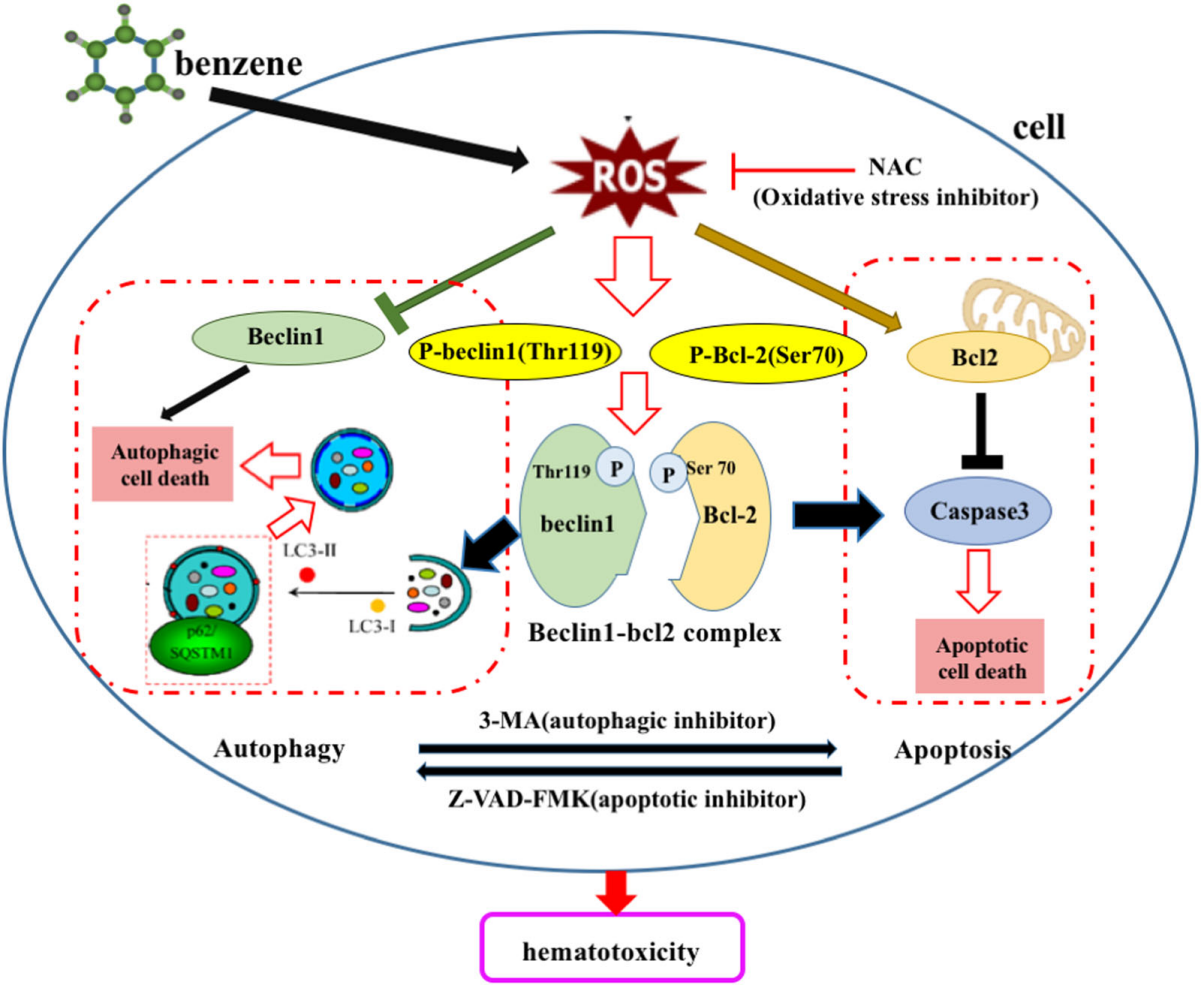
The putative schematic representation of mechanisms involved in the benzene-induced hematotoxicity

It has been reported that beclin1 binds to Bcl-2 to form beclin1-Bcl2 complex, which modulated the crosstalk between autophagy and apoptosis^24^. The relationship between beclin1 and Bcl-2 was analyzed by Bioinformatics analysis (Fig. 3s). This study aimed to investigate whether 1, 4-BQ activated autophagy and apoptosis by regulating the dissociation of beclin1-Bcl2 complex and showed that a smaller amount of beclin1 co-immunoprecipitated with Bcl-2 after 1, 4-BQ treatment (Fig. 8b). Interestingly, oxidative stress inhibitor NAC, autophagy inhibitor 3-MA and apoptosis inhibitor Z-VAD-FMK reduced the dissociation of beclin1-Bcl2 which triggered by 1, 4-BQ (Fig. 8a–c). These findings demonstrated that 1, 4-BQ promoted the dissociation of beclin1-Bcl2 complex and thereby induced autophagy and apoptosis. An overview of the exact mechanisms involved the benzene-induced hematotoxicity was presented in Fig. 9.

## Discussion

Autophagy and apoptosis are considered to be a potential toxic effect of benzene that results in cytotoxicity, proliferation, and even diseases^12,16,25^. Recent studies have reported that there are a variety of mechanisms involved in benzene-induced hematotoxicity, including autophagy and apoptosis. Our previous study has reported that benzene-induced apoptosis^11,12^, but the underlying mechanism of the relationship between autophagy and apoptosis remained unclear. In this study, we focused on investigating the specific mechanisms through which the crosstalk between autophagy and apoptosis was involved in benzene-induced hematotoxicity.

Base on previous studies, we verified that the effect of benzene on autophagy and apoptosis using TEM, Western blotting and mRFP-GFP-tagged LC3. The results showed that benzene activated autophagy, while apoptosis was induced by benzene. In addition, we further investigated benzene-induced ROS generation, which damaged cell ultrastructures and caused cell death, such as autophagy and apoptosis. It was well accepted that autophagy and apoptosis can be modulated by oxidative stress, since autophagy and apoptosis often were enhanced by oxidative stress^12,26^. In our previous study, the inhibition of 8-iso-PGF2a might be affected by inactivation of NQO1^13^. Inhibition of NQO1 has been reported to be accompanied by enhanced autophagy^14^, but the effect of NQO1 inhibition on benzene-induced autophagy remain unclear. After treating with oxidative stress inhibitor, the activation of NQO1 was shown to suppress the autophagy process which was found to aggravated benzene-induced cytotoxicity. These results suggested that benzene-induced oxidative stress enhanced autophagy.

It has been reported that benzene can led to autophagy and apoptosis^11,12,16,26^, but it is not determined whether benzene modulated the crosstalk between autophagy and apoptosis. Our results showed that both Bcl-2 and cleaved-caspase-3 were suppressed by autophagy inhibitor 3-MA, indicating the inhibition of autophagy attenuated the increase of apoptosis induced by 1, 4-BQ. When cells were treated with apoptosis inhibitor Z-VAD-FMK, autophagy markers of p62 and LC3II were totally inhibited. Further, we also found that autophagosomes (GFP+ RFP+) and autolysosomes (GFP− RFP+) was inhibited after treating with apoptosis inhibitor Z-VAD-FMK, indicating that apoptosis inhibitor Z-VAD-FMK inhibited autophagy. These results supported that there was crosstalk between benzene-induced autophagy and apoptosis.

Previously, beclin1 was shown to enhance benzene-induced autophagy^15^, but beclin1 was inhibited in 1, 4-BQ-treated cells while benzene enhanced autophagy in this study. Interestingly, we found that there was the difference of the mechanism of Beclin1-mediated benzene-induced hematotoxicity between in normal cell line and tumor cell line. We firstly found that benzene-induced the activation of autophagy in cells is not caused by the expression of beclin1, then what mechanism is responsible for the activation of autophagy? Under abnormal conditions, a balance between apoptosis and autophagy that maintains intracellular homeostasis was broken, and this balance was perturbed in neurode-generative disorders^18^. It was generally accepted that the beclin1-mediated autophagy is not only regulated by its own expression, but also based on promoting beclin1 binding to Bcl-2 to regulate the occurrence^18,19,27^. Therefore, we attempted to explore the effect of beclin-Bcl2 complex on the crosstalk between benzene-induced autophagy and apoptosis. Our data verified that benzene promoted the dissociation of beclin1 and Bcl-2, which enhanced autophagy triggered by benzene, but not the expression of beclin1.

More and more evidence about the crosstalk between autophagy and apoptosis has focused on post-translational modifications (PTMs)^17–19,24,28,29^. The phosphorylation of BH3-only domain within beclin1, or the BH3 receptor domain within Bcl-2, disrupted the beclin1-Bcl2 complex, resulting in the stimulation of autophagy^30,31^. Previous study has demonstrated that phosphorylation of the BH3-domain residue Thr119 inhibited beclin1-Bcl2 interaction^21,22,30,32^. In addition, the phosphorylation of Bcl-2 (Thr69/Ser70/Ser87) seem to be effective in abrogating beclin1-Bcl-2 complex^17,18^. However, it was unknown which site of beclin1 and Bcl-2 plays a key role in benzene-induced autophagy and apoptosis. Herein, we found that benzene-induced oxidative stress directly phosphorylated Bcl-2-Ser70 and beclin1-Thr119 dissociating the complex of beclin1-Bcl2, promoting autophagy. Autophagy was enhanced by benzene, because phosphorylation of Bcl-2 at Ser70 site and phosphorylation of beclin1 at Thr119 site is strongly induced on benzene-induced autophagy and apoptosis. It is necessary for modulating the crosstalk between benzene-induced autophagy and apoptosis. These findings provide a new insight of the mechanisms of the crosstalk between autophagy and apoptosis, which is beneficial to investigate benzene-induced hematotoxicity.

In conclusion, benzene exposure stimulated ROS generation, which in turn modulated the crosstalk between autophagy and apoptosis via phosphorylating of Bcl-2 at Ser70 site and beclin1 at Thr119 but not the expression beclin1. Meanwhile, not only was phosphorylation of Bcl-2 at Ser70 site and beclin1 at Thr119, but the autophagy was promoted by dissociation beclin1-Bcl2 complex. Therefore, benzene-induced hematotoxicity through mediating the crosstalk between autophagy and apoptosis via phosphorylation of Bcl-2 at Ser70 site and beclin1 at Thr119.

## Supplementary information

Table 1s, Table 2s

supplementary figure legends

Figure 1s

Figure 2s

Figure 3s

Figure 4s

Figure 5s
