# Supplementary material for: The crosstalk between autophagy and apoptosis was mediated by phosphorylation of Bcl-2 and beclin1 in benzene-induced hematotoxicity
Source: Cell Death Dis. 2019 Oct 10;10(10):772. doi: 10.1038/s41419-019-2004-4 (PMC6787223; doi:10.1038/s41419-019-2004-4)
Supplement: Supplementary file 1 — Table 1s, Table 2s [file 41419_2019_2004_MOESM1_ESM.docx]

**Table 1s Basic characteristics of workers, peripheral blood counts and ALT between control and benzene exposure group**

|  | | | Control (n=70) | |  | | Benzene exposure (n=70) | | | *p* | |  |
| --- | --- | --- | --- | --- | --- | --- | --- | --- | --- | --- | --- | --- |
|  | male | | | female | |  | | male | Female | |  | |
| Age | | 25-63(43.139±11.138) | | 20-65(39.000±10.364) | |  | | 21-53(32.387±8.745) | 27-48(38.000±8.718) | | <0.001* | |
| Sex (n, %) | | 35(50.000) | | 35(50.000) | |  | | 62(88.571) | 7(11.429) | | <0.001* | |
| Airborne benzene (mg/m^3^) | | 0.020-0.060  (0.049±0.012) | | 0.020-0.060  (0.051±0.008) | |  | | 0.220-6.700  (2.718±1.443) | 0.220-6.700  (2.260±2.197) | | <0.001* | |
| WBC (10^9^/L) | | 3.670-10.620  (6.248±1.435) | | 4.210-9.300  (6.178±1.305) | |  | | 3.600-9.680  (6.341±1.520) | 3.540-7.560  (5.529±1.308) | | 0.862 | |
| NEUT (10^9^/L) | | 1.940-6.800  (3.371±0.999) | | 1.990-5.250  (3.634±0.892) | |  | | 1.880-5.880  (3.496±1.098) | 1.510-5.400  (3.440±1.425) | | 0.937 | |
| PLT (10^9^/L) | | 159.000-311.000  (218.371±38.797) | | 194.000-436.100  (256.774±47.198) | |  | | 145.000-294.000  (221.679±37.040) | 160.000-284.000  (214.429±43.585) | | 0.028* | |
| Lymphocyte(10^9^/L) | | 1.260-3.410  (2.300±0.586) | | 1.18-4.170  (2.098±0.649) | |  | | 1.260-2.980  (2.043±0.464) | 0.880-2.630  (1.630±0.518) | | 0.038* | |
| ALT (U/L) | | 9.000-119.000  (30.278±19.053) | | 7.000-73.000  (18.286±12.442) | |  | | 6.000-107.000  (25.710±18.658) | 8.000-101.000  (38.286±31.894) | | 0.412 | |
| RBC(10^12^/L) | | 4.110-5.610  (4.970±0.327) | | 3.820-5.440  (4.396±0.294) | |  | | 4.370-5.740  (5.077±0.278) | 3.960-5.010  (4.521±0.327) | | <0.001* | |
| HGB(g/L) | | 131.000-177.100  (155.033±10.0294) | | 109.000-153.000  (129.114±9.209) | |  | | 134.000-173.100  (157.287±8.483) | 124.000-142.000  (134.143±7.515) | | <0.001* | |

Data are represented in the form of Min-Max(mean±SD) in this table.

*compared with control individuals, P<0.05.

**Table 2s Analysis of airborne benzene concentration, urinary t, t-MA and urinary S-PMA between two groups**

|  | Control (n=70) | Benzene exposure (n=70) | *P** |
| --- | --- | --- | --- |
|  | Median (Min, Max) | Median (Min, Max) |  |
| Airborne benzene (mg/m^3^) | 0.050 (0.020, 0.060) | 2.639 (0.220, 6.700) | **<0.001*** |
| S-PMA (ng/mL) | 0.306 (0.110, 1.380) | 0.413 (0.05, 4.950) | 0.501 |
| t, t-MA (ng/mL) | 0.118 (33.420, 221.430) | 0.119 (22.45,355.71)2 | 0.949 |

*compared with control individuals, P<0.05.
