# Supplementary material for: The crosstalk between autophagy and apoptosis was mediated by phosphorylation of Bcl-2 and beclin1 in benzene-induced hematotoxicity
Source: Cell Death Dis. 2019 Oct 10;10(10):772. doi: 10.1038/s41419-019-2004-4 (PMC6787223; doi:10.1038/s41419-019-2004-4)
Supplement: Supplementary file 2 — supplementary figure legends [file 41419_2019_2004_MOESM2_ESM.docx]

**Figures and tables of supplemental material**

**Fig. 1s The oxidative stress indexes MDA and 8-OHdG were not associated with autophagy and apoptosis.** (A-F) The correlations among MDA and 8-OHdG, autophagy and apoptosis were analyzed using correlation analysis. Data are represented in the form of mean±SD. *p<0.05 compared to control group. *p<0.05 compared to control group. ^#^p<0.05 compared to 20μM 1,4-BQ-treated group.

**Fig. 2s Autophagy-associated and apoptosis-associated proteins were not associated with all blood routine indicators.** (A-H) The correlations among autophagy-associated proteins, apoptosis-associated proteins and blood clinical parameters were analyzed using correlation analysis. Data are represented in the form of mean±SD. *p<0.05 compared to control group.

**Fig. 3s** **The relationship between beclin1 and Bcl-2 was analyzed by String software.**

**Fig. 4s 1, 4-BQ upregulated p-beclin1(Thr119).** The fluorescence intensity of p-beclin1 was measured by Image J. Data are represented in the form of mean±SD. *p<0.05 compared to control group. *p<0.05 compared to control group. ^#^p<0.05 compared to 20μM 1,4-BQ-treated group.

**Fig. 5s 3-MA and Z-VAD-FMK decreased beclin1 and Bcl-2 in 1, 4-BQ-treated cells.** (A-B) The band of beclin1 and Bcl-2 was analyzed by image J after treating with 3-MA. (C-D) The band of beclin1 and Bcl-2 was analyzed by image J after treating with Z-VAD-FMK. Data are represented in the form of mean±SD. *p<0.05 compared to control group. *p<0.05 compared to control group. ^#^p<0.05 compared to 20μM 1,4-BQ-treated group.
