# Supplementary figures and images for: The crosstalk between autophagy and apoptosis was mediated by phosphorylation of Bcl-2 and beclin1 in benzene-induced hematotoxicity
Source: Cell Death Dis. 2019 Oct 10;10(10):772. doi: 10.1038/s41419-019-2004-4 (PMC6787223; doi:10.1038/s41419-019-2004-4)

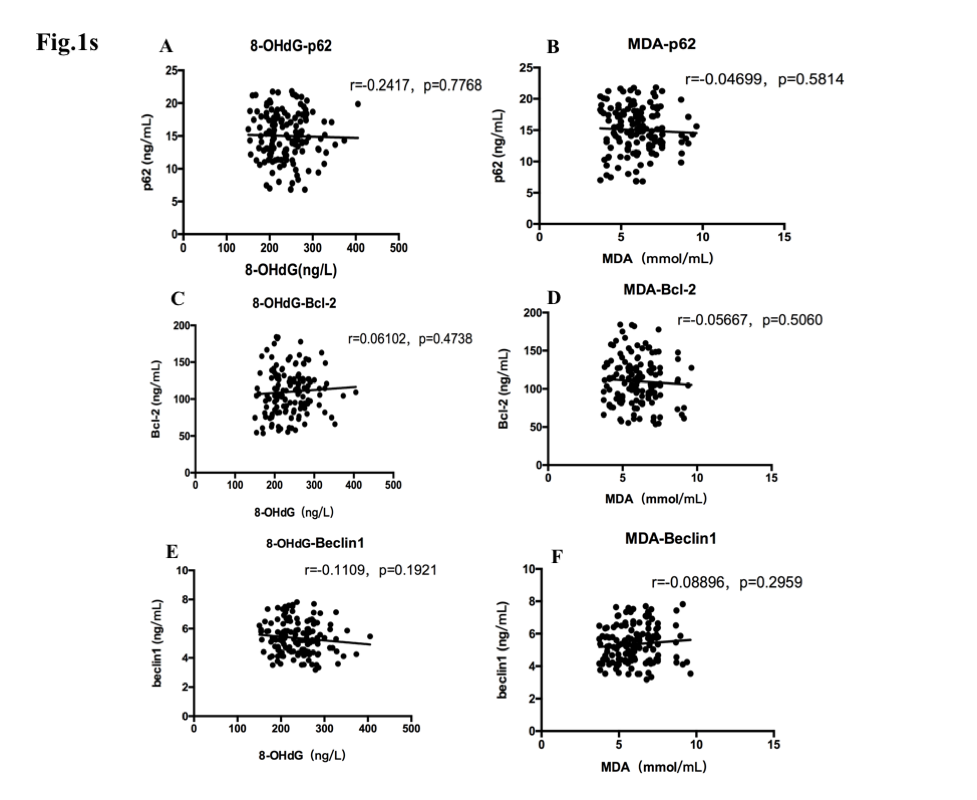

Supplement: Supplementary file 3 — Figure 1s [file 41419_2019_2004_MOESM3_ESM.tif]

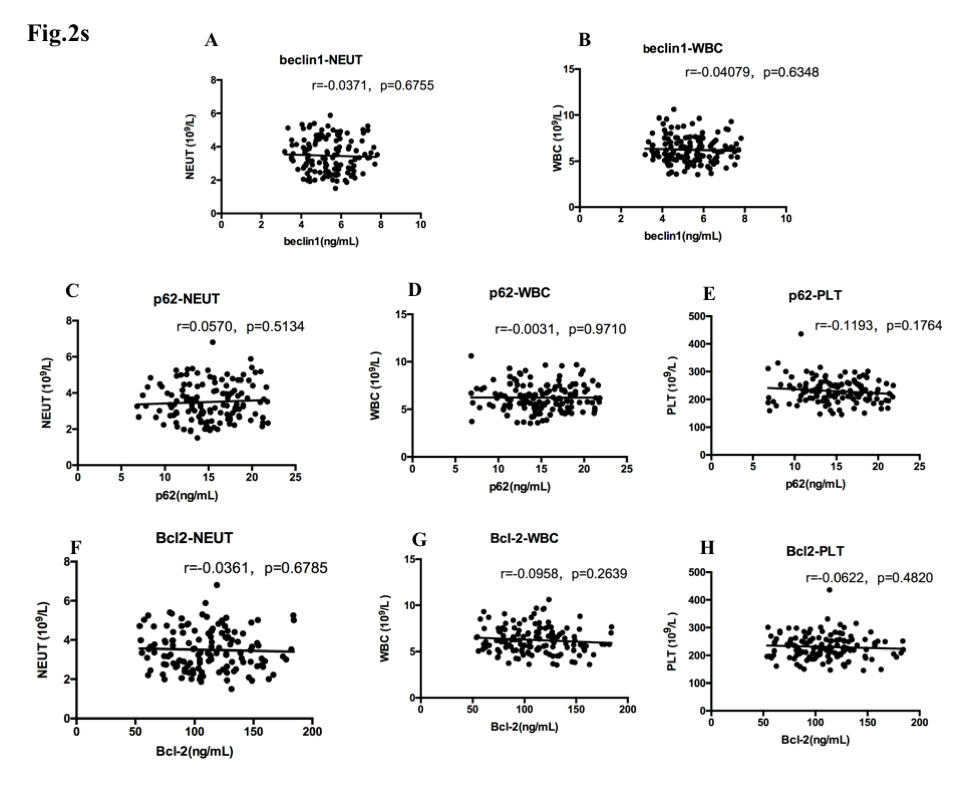

Supplement: Supplementary file 4 — Figure 2s [file 41419_2019_2004_MOESM4_ESM.tif]

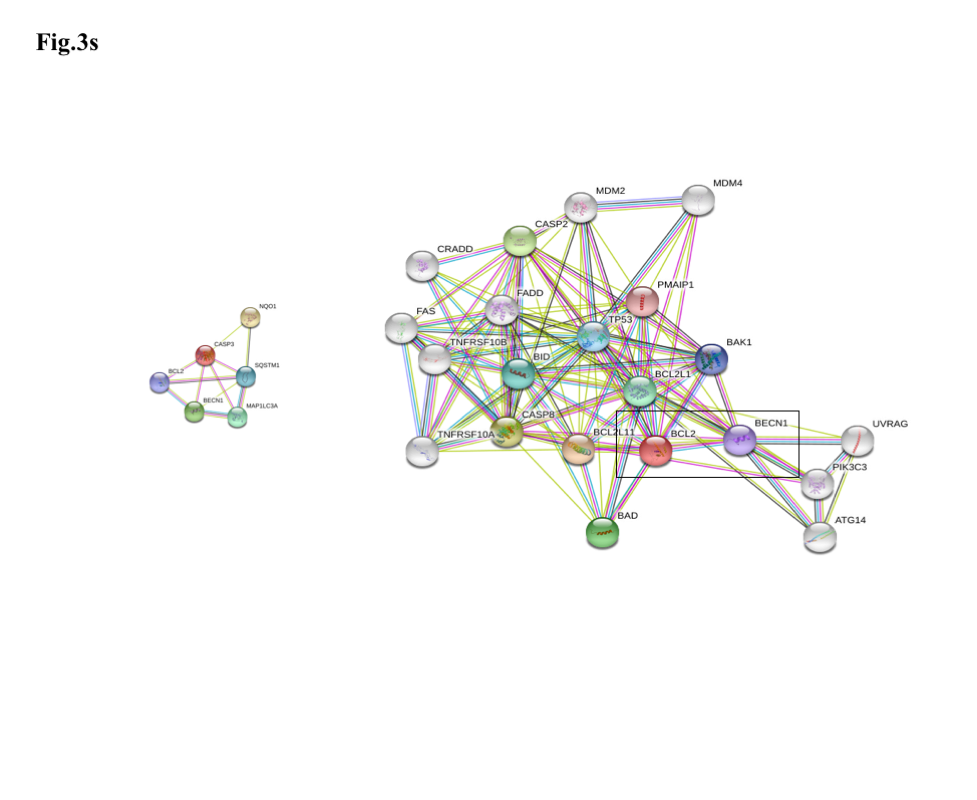

Supplement: Supplementary file 5 — Figure 3s [file 41419_2019_2004_MOESM5_ESM.tif]

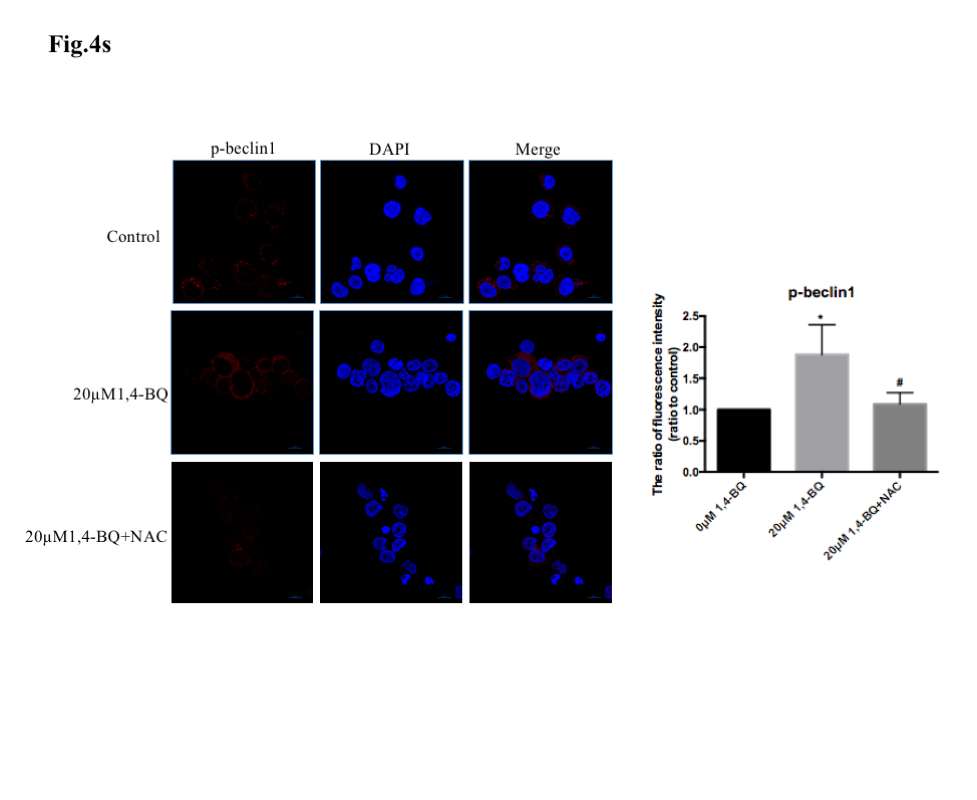

Supplement: Supplementary file 6 — Figure 4s [file 41419_2019_2004_MOESM6_ESM.tif]

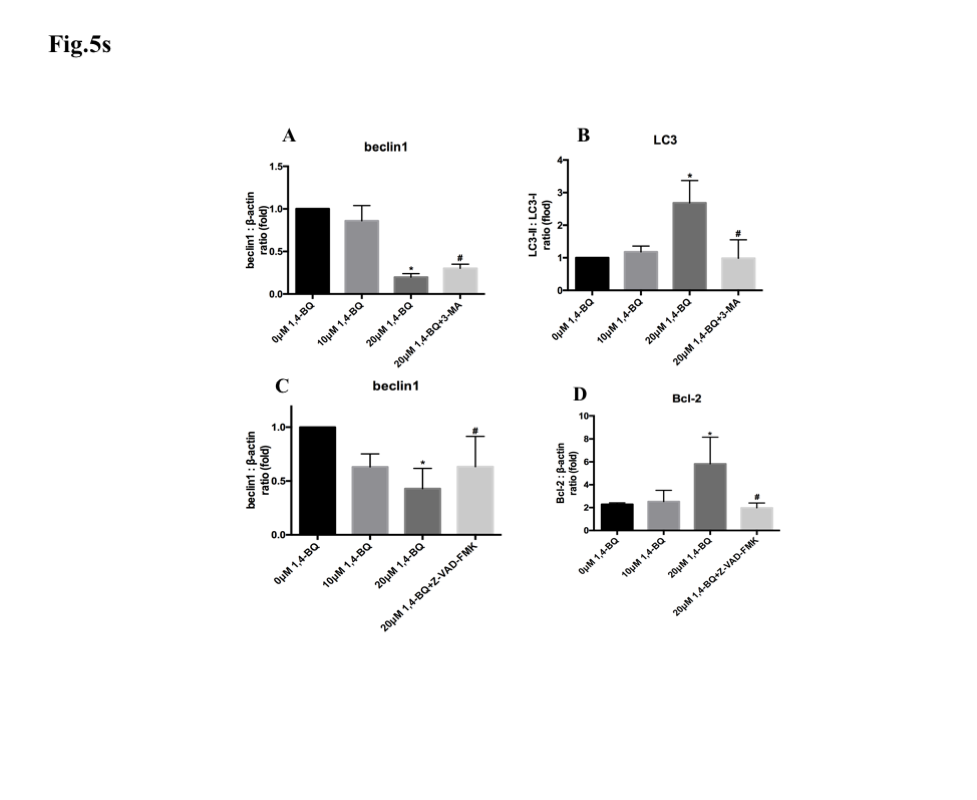

Supplement: Supplementary file 7 — Figure 5s [file 41419_2019_2004_MOESM7_ESM.tif]
